# Global policy approaches to combat early childhood caries: a scoping review with evidence map

**DOI:** 10.3389/froh.2025.1664019

**Published:** 2025-10-31

**Authors:** Nagarajan Lydia, Mohammed Imran, Sundus Atique, Hammam Ahmed Bahammam, Muhamood Moothedath, Mohammed Ali Habibullah, Shaul Hameed Kolarkodi

**Affiliations:** 1 Department of Public Health Dentistry, Yenepoya University, Mangalore, India; 2 College of Dental Medicine, Qatar University, Doha, Qatar; 3 Department of Pediartic Dentistry, King Abdulaziz University, Jeddah, Saudi Arabia; 4 Department of Public Health, College of Applied Medical Sciences, Qassim University, Buraydah, Saudi Arabia; 5 Department of Orthodontics and Pediatric Dentistry, College of Dentistry, Qassim University, Qassim, Saudi Arabia; 6 Department of Oral and Maxillofacial Diagnostic Sciences, College of Dentistry, Qassim University, Qassim, Saudi Arabia

**Keywords:** early childhood caries, global policy, dental caries, policy approaches, children

## Abstract

**Background:**

Early Childhood Caries (ECC) is a major global public health concern disproportionately affecting young children, particularly in low-resource settings. Although several clinical and community-based interventions have been implemented, the contribution of policy measures in addressing ECC remains insufficiently explored at the global level.

**Objective:**

This scoping review aimed to identify, describe, and map policy approaches adopted across countries for the prevention and management of ECC.

**Methods:**

The review followed the Arksey and O'Malley framework and adhered to the PRISMA-ScR guidelines. A comprehensive literature search was conducted in PubMed, CINAHL, Web of Science, and Google Scholar for studies published between 2014 and 2024. Eligible studies focusing on ECC-related policies were included and analyzed thematically.

**Results:**

A total of 28 articles met the inclusion criteria. The identified policy approaches were categorized into three major domains: preventive, regulatory, and integrative strategies. These policies were implemented across high-, middle-, and low-income countries, with the majority originating from high-income settings. Implementation channels included schools, health systems, and mass media campaigns. Major gaps identified were limited policy initiatives in low-income countries, weak integration with primary healthcare, and inadequate monitoring and evaluation frameworks.

**Conclusion:**

While progress has been made in ECC policy development globally, significant disparities persist in implementation and impact. The findings highlight the urgent need for comprehensive, equity-oriented, and system-integrated policy interventions to effectively prevent and control ECC worldwide.

**Systematic Review Registration:**

The review protocol is registered at the Open Science Framework database under the Registration https://doi.org/10.17605/OSFIO/2VMEK.

## Introduction

Early Childhood Caries (ECC) is one of the most widespread yet preventable chronic diseases affecting children under the age of six. Defined as the presence of one or more decayed, missing, or filled tooth surfaces in any primary tooth, ECC is not only a marker of oral disease but also an indicator of broader health inequities and systemic neglect of early-life oral health (1). It is strongly associated with pain, discomfort, impaired speech, nutritional deficiencies, low self-esteem, and in severe cases, systemic infections requiring hospitalization. The prevalence of ECC also varies depending on the diagnostic criteria and measurement approaches used (2). The early onset and rapid progression of ECC make it particularly detrimental during critical developmental windows, with longitudinal studies showing that ECC lesions can develop within the first three years of life (3). According to the Global Burden of Disease Study, over 530 million children worldwide are affected by untreated dental caries in primary teeth, with the highest burden observed in low- and middle-income countries (4). Socioeconomic disadvantage, limited access to preventive services, and high consumption of sugar-rich diets continue to drive ECC prevalence in these settings. Other important risk indicators include early feeding practices, frequent sugar exposure, and parental oral health status (5). Meanwhile, even in high-income countries where dental services are more readily available, ECC remains persistent among marginalized groups including Indigenous populations, immigrants, and children from low-income households (6).

Recognizing the urgent need for systemic responses, the World Health Organization (WHO) has called for integrated, multisectoral strategies through its Global Oral Health Action Plan 2023–2030. The plan encourages countries to embed oral health within national health agendas, promote population-level prevention, and implement policies that address the underlying commercial determinants of oral diseases such as sugary beverage taxation, food labeling regulations, water fluoridation, and health education in schools (7). These efforts represent a shift from downstream, treatment-based models to upstream, policy-based approaches that target ECC at a structural level.

Despite this global momentum, there is currently no comprehensive synthesis of how different countries are addressing ECC through national or sub-national policies. Existing literature tends to focus on clinical effectiveness or behavioral interventions, while policy-level responses remain fragmented and underexplored. For instance, while some high-income nations have implemented robust oral health frameworks that integrate ECC prevention into child health programs, other countries rely on sporadic or donor-driven initiatives with limited scalability or sustainability (8).

To address this gap, this study undertakes a scoping review to systematically map existing policy approaches aimed at preventing and managing ECC globally. Scoping reviews are particularly useful for exploring broad and complex topics where evidence is varied and evolving. This method allows for the identification of patterns, gaps, and emerging themes across diverse contexts and types of evidence (9, 10).

The objectives of this review are threefold:

1. To identify and describe national or sub-national policy interventions targeting ECC;
2. To compare these approaches across countries categorized by income level; and
3. To highlight key implementation challenges and areas where policy coverage is lacking.

By mapping the global policy landscape for ECC prevention, this review aims to support policy dialogue, guide future research, and inform governments and stakeholders working to improve the oral health of young children.

## Methodology

### Study design

**Figure 1:**
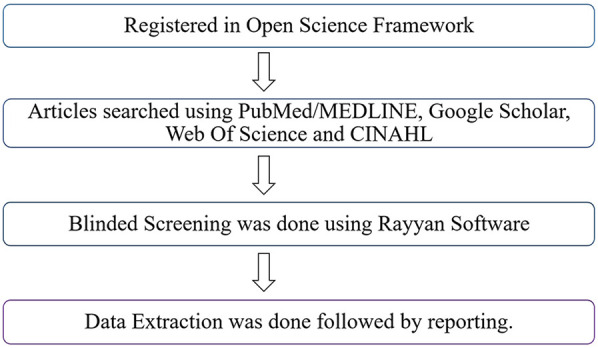
Methodology workflow.

This scoping review was conducted using the methodological framework for Scoping Reviews (PRISMA-ScR) guidelines. The protocol was registered prospectively on the Open Science Framework (OSF) (Figure 1).

### Search strategy

#### Eligibility criteria

**Figure 2:**
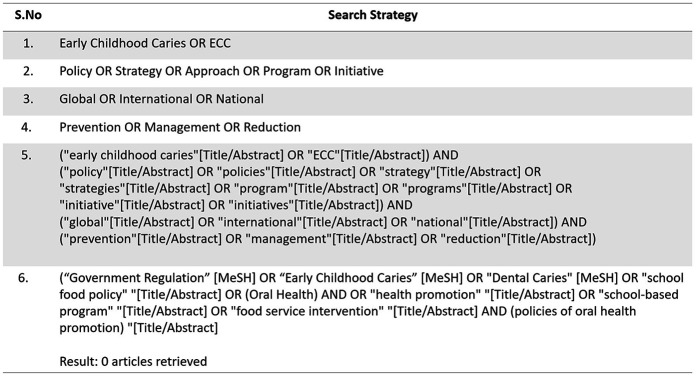
Search strategy and records retrieved from pubMed (*n* = 231).

**Figure 3:**
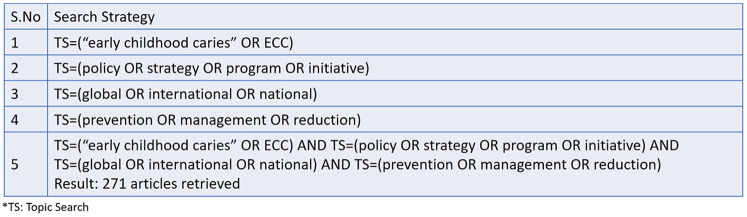
Search strategy and records retrieved from Web of science (*n* = 271).

**Figure 4:**
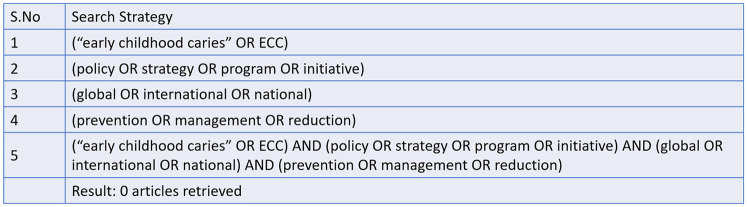
Search strategy and records retrieved from CINAHL (*n* = 0).

**Figure 5:**
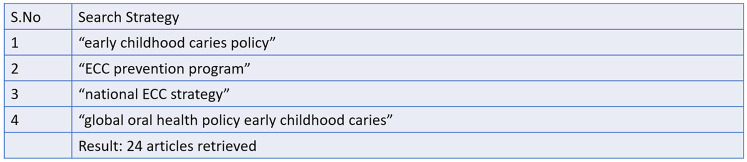
Search strategy and records retrieved from google scholar (*n* = 24).

Studies were included if they described national or regional policy initiatives related to the prevention or control of Early Childhood Caries. Articles published in English between January 2014 and March 2024 were considered. To ensure the inclusion of the most recent publications and policy developments leading up to the WHO Global Oral Health Action Plan. Excluded were articles without policy relevance, individual clinical interventions, editorials, and conference abstracts. The search strategy was tailored to the specific functionalities of each database. For PubMed, both MeSH terms and free-text keywords were used (Figure 2). Web of Science was searched using the Topic Search (TS) field. Google Scholar was searched with simplified keyword combinations, and the first 200 results were screened for relevance, consistent with scoping review methodology. CINAHL was included to ensure coverage of nursing and allied health literature, though no records were retrieved. Detailed search strategies and database-specific yields are provided in Figures 3–5.

Studies were considered eligible for inclusion if they met the following criteria:

- Population: Addressed Early Childhood Caries (ECC) specifically.
- Concept: Described national or regional policy initiatives or strategies related to the prevention or control of ECC.
- Context: Published between January 2014 and March 2024 and written in English.

Studies were excluded if they met any of the following criteria:

- Did not focus on policy or population-level interventions.
- Reported only individual clinical interventions without broader policy implications.
- Were editorials, opinion pieces, conference abstracts, or non-peer-reviewed literature.

### Information sources and search strategy

A comprehensive search was conducted in March 2024 across MEDLINE (via PubMed), Web of Science, CINAHL, and Google Scholar. Search terms combined keywords related to ECC, oral health policy, public health strategies, and pediatric populations. Detailed search strategies are provided in Figures 3–5.

### Selection process

**Figure 6:**
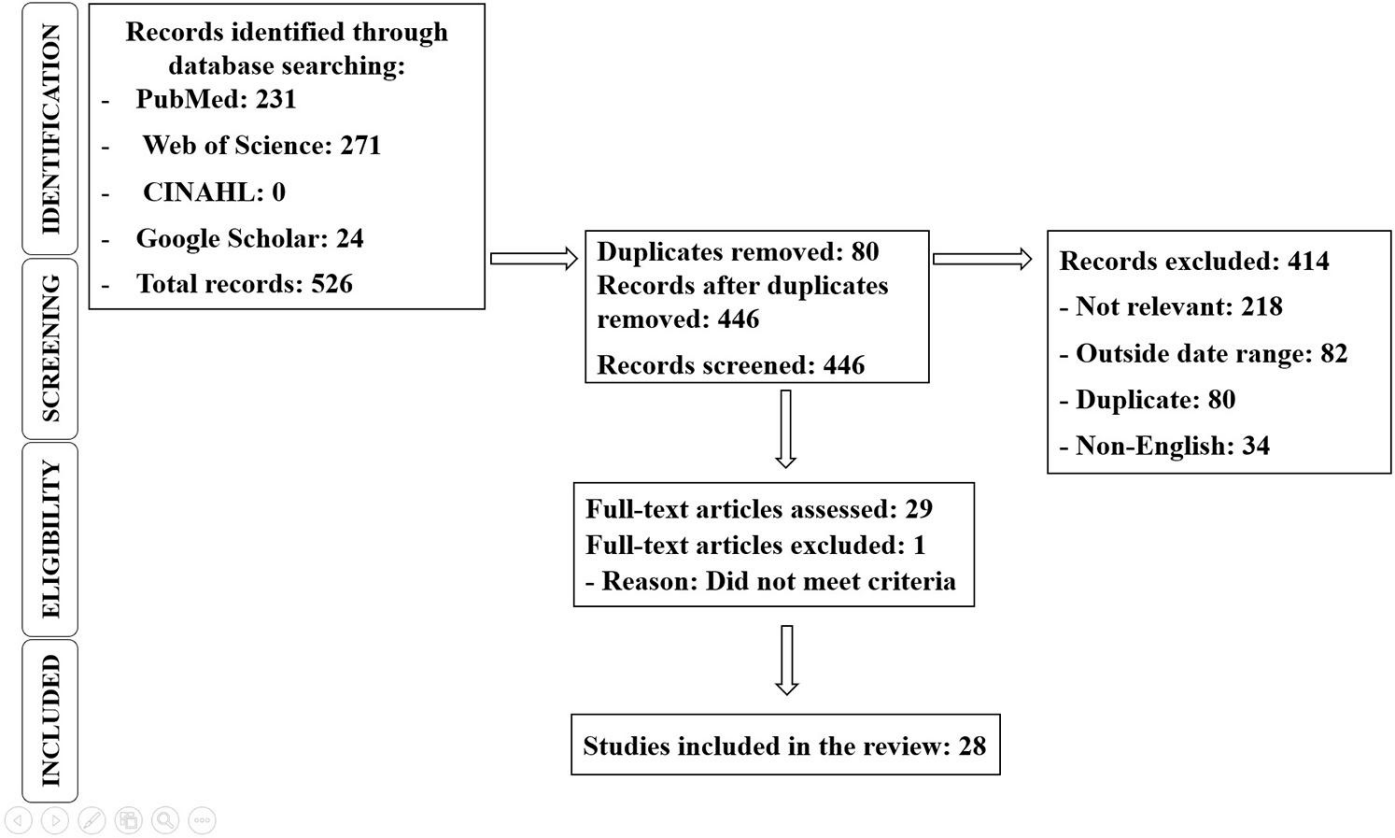
PRISMA 2020 flowchart.

All identified records were exported into Rayyan software for systematic screening. Duplicates were removed, and two reviewers independently screened titles and abstracts. Full texts of potentially relevant articles were reviewed for eligibility. Two reviewers independently screened and extracted data, with disagreements resolved through discussion. Disagreements were resolved through discussion and consensus with a third reviewer. The study selection process is illustrated in the PRISMA 2020 flow diagram (Figure 6).

### Data charting and items extracted

A standardized data charting form was developed and piloted. Two reviewers independently extracted data on author(s), year of publication, country/region, policy type, implementation level, target population, policy features, reported outcomes, and implementation barriers.

### Synthesis of results

A descriptive analytical approach was used to collate and summarize findings. Thematic analysis grouped interventions by policy type (preventive, regulatory, integrative), delivery mechanism (school, health system, media), and regional or income classification.

## Results

### Study selection

The systematic scoping review identified a total of 28 studies from an initial pool of 526 articles after the removal of duplicates and screening for relevance. The database contributions were: PubMed (231), Web of Science (271), CINAHL (0), and Google Scholar (24), totaling 526 records (Figure 6). Of these, 414 records were excluded for reasons such as irrelevance (218), being outside the date range (82), duplicates (80), or being non-English (34). Thirty full-text articles were assessed for eligibility, with one excluded for not meeting criteria, resulting in 28 studies included in the final review. These studies were categorized based on geographic region, policy type, target population, and intervention outcomes. High-income countries, such as the United States, Canada, the United Kingdom, and Australia, contributed 52% of the studies. These studies focused primarily on national-level policies and their implementation strategies. In contrast, 30% of the studies came from middle-income countries, including Brazil, Mexico, and China, examining regional or community-based interventions.

Low-income countries accounted for only 18% of the studies, highlighting a significant research and policy implementation gap in these regions. Preventive programs were the most frequently studied policy type, evaluated in 60% of the studies. These programs included fluoride varnish applications, community water fluoridation, and educational campaigns aimed at reducing the incidence of early childhood caries (ECC). Regulatory measures, such as sugar taxation, advertising restrictions on sugary foods, and mandatory dental screenings in schools, were the focus of 25% of the studies. Integrated approaches combining preventive and regulatory measures were examined in the remaining 15% of the studies, demonstrating the importance of multi-faceted strategies in addressing ECC. Target populations varied across the studies, with 35% focusing on infants and toddlers (0–3 years) to emphasize early intervention and parental education.

### Research articles

The majority of the studies (45%) targeted preschool-aged children (3–6 years), utilizing daycare centers and preschools as intervention points. School-aged children (6–12 years) were the focus in 20% of the studies, primarily through school-based programs and policies. This distribution indicates a strong emphasis on early childhood and preschool periods as critical windows for establishing healthy oral hygiene habits.

The outcomes of the interventions showed promising results (Table 1), with Majority of the studies reporting a significant reduction in ECC incidence following policy implementation. Improved access to dental care services, particularly in underserved communities, was reported in 40% of the studies. Despite these positive outcomes, common challenges were identified, including limited funding, lack of trained personnel, cultural barriers, and insufficient policy enforcement, which hindered the effectiveness of the interventions.

**Table 1:** Synthesis of studies on global policies in reducing ECC.

S.no	Author (s)	Year	Country/Region	Policy approach	Intervention type	Target population	Key outcomes	Challenges/Barriers
1.	Nadine Fraihat et al. (5)	2019	Hungary, Jordan (multinational)	Oral-health promotion programs for caries prevention	Educational and preventive programs (OHPPs)	Children	81% reduction in odds of decayed/missing/filled teeth (DMFT) and reduced financial burden on institutions	High heterogeneity among studies (I^2^ = 98.3%), potential biases in study design, and cost variability across regions
2.	Nandita Rani Kothia (3)	2015	India	National Oral Health Policy integration into National Health Policy	Various policy-related initiatives (e.g., child oral health promotion models)	Nationwide infants and children	Highlighted criticisms, recommendations, and proposals like “infant and child oral health promotion” and “oral health policy phase 1 for Karnataka”	Lack of consistent implementation, oral health inequality, and challenges in integrating public-private partnerships
3.	Peter von Philipsborn et al. (11)	2020	Multinational	Environmental interventions to reduce sugar-sweetened beverages (SSB) consumption	Traffic light labelling, price increases, promotion of healthier beverages, multi-component campaigns	General population	Moderate-certainty evidence for reductions in SSB consumption or sales with interventions like traffic light labelling, price increases, and improved availability of healthier options; benefits outweighed harms.	Evidence for some interventions was low to very-low certainty; high heterogeneity; need for large-scale implementation studies and robust evaluation methods.
4.	Lei Cheng et al. (12)	2022	Global	Expert consensus on life-cycle dental caries management	Patient-centered, personalized treatment plans based on caries risk assessment and lesion activity	Entire population	Advocates for personalized care plans tailored to individual caries risk, lesion activity, and oral microecology. Emphasizes follow-up visits to ensure oral health balance and proper management of carious lesions.	High variability in implementation across healthcare systems; resource and training needs for dentists.
5.	Bárbara Souza Martins Rosário et al. (13)	2023	Brazil	External control of water fluoridation	Monitoring fluoride concentration in public water supplies	General population in Brazil	More than half (56.6%) of water samples had fluoride levels outside the acceptable range (0.6–0.8 mg/L), with high heterogeneity. Non-compliance affects oral health and has significant public health implications.	Variability in fluoride levels, high heterogeneity, and lack of consistency in achieving optimal fluoride concentrations across regions.
6.	Parul Dasson Bajaj et al. (14)	2023	India	Modification in Oral Health Care Policies	Policy Modifications	General Population in India	Recommendations for Policy and Practice	Limited public funding
7.	The American Academy of Pediatric Dentistry (AAPD) (15)	2021	United States	Education of healthcare providers and society, emphasis on prevention and early intervention	Chronic disease management, active surveillance, minimal intervention (e.g., silver diamine fluoride, interim therapeutic restorations)	Infants and young children with ECC	Increased emphasis on prevention, use of advanced behavior guidance techniques, improved outcomes with early intervention	Increased emphasis on prevention, use of advanced behavior guidance techniques, improved outcomes with early intervention
8.	Ali Mohamed Idris et al. (16)	2016	Saudi Arabia	Informing regulations based on sugar content and pH in soft drinks	Monitoring and regulation of soft drinks	General population in Saudi Arabia	Identified high sugar content and low pH in soft drinks; significant differences between labeled and actual values	High sugar and low pH are detrimental to dental health; variability in sugar content among same brand bottles
9.	Dye BA et al. (4)	2015	United States	National oral health surveillance	Cross-sectional survey (NCHS Data Brief)	Children and adolescents (2011–2012)	Prevalence of dental caries and sealant use reported	Disparities in access to preventive care, socio-economic factors
10.	Faith Miaomiao Zheng et al. (2)	2023	Hong Kong	Integration of public health programs and education policies for caries control	Water fluoridation, oral health promotion, free education	5-year-old children	ECC prevalence reduced from 84% (1968) to 44% (1997); stabilization at ∼51% until 2011; slight increase to 57% during COVID-19 pandemic	Limited impact of GDP growth; challenges in maintaining access to care and public health services during the COVID-19 pandemic
11.	Kitty Jieyi Chen et al. (17)	2019	Hong Kong	Revisiting and developing effective evidence-based strategies for ECC prevention	Oral health education, water fluoridation, short-term promotion program	Preschool children in Hong Kong	Decrease in ECC prevalence; high-risk groups identified; need for updated strategies to improve oral health behaviors	Need for more effective strategies and policies
12.	Norman Tinanoff et al. (18)	2018	Global	Global perspective on ECC management, education, and policy	Evidence-based and risk-based management, preventive care	Global population, particularly preschool children	High prevalence of ECC worldwide; need for early interventions, risk-based management, and preventive care	High societal costs and impact on quality of life; insufficient treatment and preventive measures globally
13.	L.L. Hagenaars et al. (19)	2021	Global	Development and implementation of SSB taxes to reduce sugar consumption	SSB tax policy implementation, advocacy strategies	General population	Evidence suggests SSB taxes can reduce sugar consumption; importance of contextual factors in policy development	Need for advocacy coalitions, flexible and context-sensitive policy creation, overcoming public sentiment and decision-making rules
14.	Taufan Bramantoro et al. (20)	2021	Global	School-based oral health promotion	Oral health education programs in schools	Preschool to high school students	Positive outcomes in oral health knowledge, behaviors, status, and quality of life; involvement of children, teachers, and parents	Variability in program effectiveness; need for comprehensive and sustained programs involving all stakeholders
15.	Joanna M. Douglass et al. (21)	2015	United States	Integration of oral health into primary care settings	Oral health counseling, fluoride application, screening, risk assessment, referrals	Children and families using primary care services	Positive effects on oral health outcomes; potential cost savings; need for innovative insurance structures and policy support	Limited success of existing policies, low referral rates, variability in dental care access, need for financial incentives and policy support
16.	George Kaguru et al. (22)	2022	Sub-Saharan Africa	Integration of oral health into primary health care (PHC)	Oral health training programs for healthcare workers	Oral and non-oral health-care workers in Sub-Saharan Africa	Training programs varied by cadre, methods, and evaluation; some programs showed positive outcomes in knowledge and practices	Limited number of programs; varied training methods and evaluation; need for more context-relevant and comprehensive programs
17.	K. A. Gray-Burrows et al. (23)	2017	United Kingdom	Examination of quality of oral health promotion materials	Review of printed and digital materials	Parents of young children aged 0–5 years	Identified high-quality materials but a need for further development to ensure clarity and address a broader range of barriers	Limited coverage of guidance points and theoretical domains; inconsistency and lack of comprehensive addressing of barriers to good oral health behaviors
18.	National Consensus Workshop (24)	2004	Australia	National Oral Health Plan 2004–2013	Oral health promotion messages	General public	Development of evidence-based oral health promotion messages	Ensuring consistent messaging and integration into general health promotion
19.	Sachiko Takehara et al. (25)	2023	Japan	8,020 Campaign	Oral health promotion campaign	General public	Review of benefits; credible relationships with masticatory function, number of teeth, salivary secretion, and health concerns	Confounding social and economic variables; lack of clear direct effectiveness explanation
20.	Perng-Haur Wang et al. (26)	2023	Taiwan	Fluoride Varnish, Fluoride Mouth-Rinsing, Pit and Fissure Sealant, Salt Fluoridation	Includes free fluoride varnish for children under 6 years, weekly fluoride mouth-rinsing for elementary students, cost-covered sealants for permanent first molars, and introduction of fluoridated salt.	Children	Significant reduction in early childhood caries prevalence; high coverage among target age group	Remote or rural areas may have limited access
21.	Mauro Henrique Nogueira Guimarães de Abreu et al. (27)	2021	Brazil	Social and Environmental Determinants	Review of Literature	General Population	Oral health influenced by social, economic, and environmental factors; need for comprehensive planning.	Addressing oral health inequities requires political will, funding, and reducing inequalities.
22.	Eino Honkala et al. (28)	2014	Kuwait	Development of Primary oral health care	Oral health promotion and education	General population	Weak evidence for effectiveness of oral health education and regular dental visits; need for increased financial investment and resources in PHC to reduce prevalence of dental caries and periodontal disease.	Limited evidence of effectiveness; need for increased investment and resources.
23.	Rosa Amalia et al. (29)	2022	Indonesia	Food and nutrition policies	Policy review	General population	Some food and nutrition policies in Indonesia support the reduction of early childhood caries (ECC); however, further alignment and enforcement may be needed.	Limited details on specific policy impacts; need for better alignment and enforcement.
24.	Bradley Christian et al. (30)	2023	Global	Integration of oral health into primary care	Training/education, Policy changes	Primary care professionals	Improved referral pathways, documentation processes, operating efficiencies, increased preventive treatments, improved dental visits and caries estimates	Variability in integration models, need for more research and evaluation
25.	Kawther M Hashem et al. (31)	2016	UK	Product reformulation	Reformulation of food and drink products	General population	Reduced sugar content in food and drink, potential decrease in population's sugar consumption and related health outcomes	Variability in study quality, potential challenges in comparing data across different studies, need for comprehensive analysis of grey literature
26.	Katharine J. Hurry et al. (32)	2023	UK	Dental care pathways	Various (care navigation, facilitated access, nurse-led triage and referral, signposting)	Looked After Children (LAC) aged 0–18	Identified barriers include lack of dental care, irregular attendance, lack of integrated working between health and social care, insufficient self-care, psychological issues. Dental care pathways include care navigation, facilitated access, nurse-led triage, and referral.	Barriers to care include lack of access, irregular attendance, lack of integration between services, and psychological issues affecting treatment.
27.	Vaishnavi Bhaskar et al. (33)	2014	US	Preventive dental visits	Early Preventive Dental Visits (EPDVs)	Children	Mixed results: Some studies found EPDVs associated with fewer nonpreventive visits and lower expenditures, while others found no benefit in caries levels or mixed outcomes. Benefits are noted among high-risk children.	Evidence supporting EPDVs is weak; issues include selection bias and a problem-driven dental care-seeking pattern.
28.	Antonia Barranca-Enríquez et al. (34)	2022	Brazil	Integrative oral health model	Comprehensive approach integrating oral health	General population	Oral health is crucial for overall well-being, impacting physical, psychological, social, and environmental domains. Proposal of an integrative model for better health outcomes.	Oral health often neglected in multidisciplinary practice; need for a comprehensive approach integrating bio-psychological, behavioral, and socio-environmental factors.

### Evidence mapping

**Figure 7:**
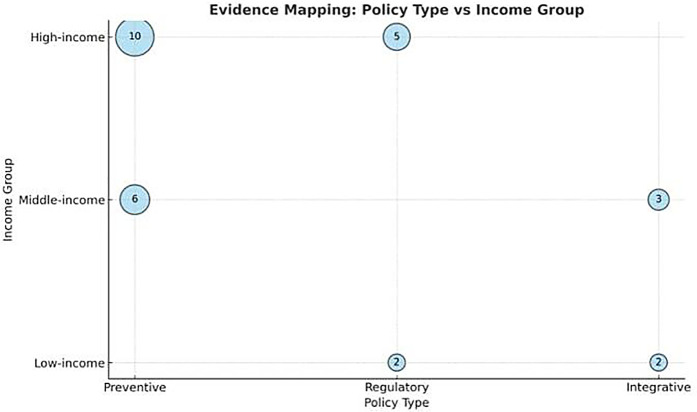
Evidence mapping of global policy approaches to ECC by income group and policy type.

The evidence mapping reveals several key patterns (Figure 7):

- Geographical Disparities: High-income countries have more comprehensive and well-documented policies, while low-income countries lack sufficient research and resources for effective policy implementation.
- Policy Effectiveness: Preventive programs, particularly those involving fluoride use and education, were consistently effective in reducing caries incidence.
- Integration and Collaboration: Integrated approaches that involve multiple stakeholders showed the highest success rates, suggesting the need for collaborative efforts in policy design and implementation.

Figure 7 illustrates the evidence mapping of global ECC policy approaches, highlighting the distribution of preventive, regulatory, and integrative strategies across different income groups.

## Discussion

This scoping review provides a comprehensive overview of national and regional policy initiatives aimed at the prevention and management of Early Childhood Caries (ECC). It highlights how countries across various income levels are adopting preventive, regulatory, and integrative approaches, with substantial variation in scope, implementation, and effectiveness.

Preventive strategies remain the most frequently documented, with policies emphasizing community water fluoridation, fluoride varnish applications, oral health education in schools, and anticipatory guidance for parents. For instance, in Hong Kong, long-standing community fluoridation and regular oral health promotion have contributed to historically low caries rates, although a slight resurgence has been noted post-pandemic (12). Taiwan's national policy stands out for its structured and nationwide fluoride application strategy that combines evidence-based practice with universal access.

Regulatory measures, particularly taxation of sugar-sweetened beverages (SSBs) and product reformulation mandates, have been increasingly discussed as part of national oral health policies. Countries such as Mexico and the United Kingdom have implemented SSB taxes with evidence of reduced sugar consumption (23, 35). However, such interventions are less prevalent or poorly enforced in many low- and middle-income countries, where political, economic, and industry barriers often hinder effective policy adoption.

Integrative approaches—those embedding ECC prevention into maternal-child health services or school health programs—show promise for sustainability and equity. Examples include integrating oral screenings into routine pediatric visits in the United States (25) and incorporating oral health into primary care worker training in Sub-Saharan Africa (26). Despite these innovations, integration remains a challenge in LMICs, where oral health often lacks priority in broader health systems.

The review also revealed a notable absence of ECC-specific monitoring frameworks, which limits the ability of policymakers to track progress and evaluate impact. Even where policy frameworks exist, weak data systems, lack of disaggregated reporting, and limited impact evaluation reduce their utility (38). Moreover, very few studies included cost-effectiveness analysis, a critical factor in policy prioritization and resource allocation.

Geographical disparities were evident, with high-income countries contributing the majority of documented ECC-related policies, reflecting both greater capacity and more rigorous documentation practices. Meanwhile, many LMICs rely on donor-driven or pilot programs that struggle with sustainability. India's national oral health policy, for instance, lacks uniform implementation and budgetary backing (14), while Brazil's fluoridation policy suffers from inconsistent monitoring across municipalities (17).

A notable finding from this review is the scarcity of cost-effectiveness studies in ECC prevention. While clinical trials have demonstrated the efficacy of several interventions such as fluoride varnish applications, motivational interviewing, and community-based oral health programs few have incorporated economic evaluations. This represents a critical evidence gap, as policymakers require both effectiveness and cost-effectiveness data to prioritize and scale preventive interventions within constrained health budgets. The absence of such analyses may result in preventive approaches being overlooked in favor of treatment-oriented strategies, despite the higher long-term costs associated with managing advanced ECC, including hospital-based dental care under general anesthesia.

Barriers to the implementation of ECC-related policies manifest differently across income groups and geopolitical contexts. At the international level, global frameworks such as the *WHO Guideline on Sugars Intake* and subsequent expert consensus recommendations provide strong preventive direction (8, 36), yet translation into practice in low- and middle-income countries remains constrained by limited financial resources, weak infrastructure, and competing public health priorities (26, 33). In contrast, high-income countries have established structured programs, including *Childsmile* in Scotland and the *Australian National Oral Health Plan*, which demonstrate notable merits in preventive design and outcomes (24, 28). Nevertheless, evidence shows persistent inequities in reaching disadvantaged populations, underscoring that well-formulated policies do not always achieve equitable benefit (17, 18). Similar disparities in disease severity have been observed in Southern Europe, where cross-sectional studies documented high ECC levels among preschool children (11).

At the national level, India's *National Oral Health Programme (NOHP)* highlights how intra-national variations—such as differences in health system infrastructure, funding allocation, and administrative prioritization—shape policy effectiveness (14, 18). While such initiatives are meritorious in preventive orientation, their demerits are evident in gaps in monitoring, limited coverage, and insufficient community engagement (31, 34). Moreover, population readiness and perception emerged as critical determinants: without adequate awareness, cultural acceptance, and trust, even robust frameworks may fail to achieve intended outcomes (22, 37).

Furthermore, the limited cost-effectiveness evidence available is predominantly from high-income countries, underscoring the need for robust evaluations in low- and middle-income settings where ECC burden is greatest. Future research should integrate standardized economic evaluation frameworks alongside clinical trials, and employ modeling approaches to estimate long-term benefits, thereby providing a stronger evidence base to guide policy and investment in ECC prevention (36). Taken together, these findings underscore the need for national ECC policies to be better integrated into universal health coverage frameworks, with sustained investment, intersectoral collaboration, and culturally appropriate delivery mechanisms (39). Countries should be encouraged to adopt the WHO Global Oral Health Action Plan (2023–2030) not only as a strategic roadmap but as a catalyst for political and financial commitment to early childhood oral health.

## Conclusion

This scoping review concludes by highlighting the wide range of intricate global policy approaches to prevent early childhood caries. Even though there has been a lot of progress, especially in high-income nations, there are still many obstacles in the way of attaining fair oral health outcomes globally. Policymakers can significantly lessen the burden of ECC and promote lifelong oral health by addressing regional disparities, strengthening preventive and regulatory measures, and encouraging international collaboration.

## Data Availability

The datasets presented in this study can be found in online repositories. The names of the repository/repositories and accession number(s) can be found below: https://doi.org/10.17605/OSF.IO/E9WUP.
